# The mesencephalic trigeminal neuron: electrophysiological insights into function and dysfunction

**DOI:** 10.3389/fncel.2026.1752701

**Published:** 2026-03-10

**Authors:** Soju Seki, Akifumi Enomoto, Susumu Tanaka

**Affiliations:** 1 Department of Oral and Maxillofacial Surgery, Graduate School of Dentistry, The University of Osaka, Suita, Osaka, Japan; 2 Department of Oral and Maxillofacial Surgery, Kindai University, Faculty of Medicine, Sakai, Japan

**Keywords:** electrophysiology, mesencephalic trigeminal neuron, MTNs, patch clamp, primary afferent neurons, proprioception, VMEs, voltage-dependent sodium channel

## Abstract

Mesencephalic trigeminal neurons (MTNs) are the sole primary afferent neurons with cell bodies located within the central nervous system. MTNs convey proprioceptive inputs from masticatory muscles and periodontal ligaments, thereby contributing to the precise regulation of jaw–oral motor functions. Through ionic mechanisms such as currents generated by the voltage-dependent sodium (Nav) channel isoform Nav1.6, hyperpolarization-activated currents, and persistent inward currents, MTNs generate sustained and burst firing that regulate masticatory rhythm and jaw-jerk reflex timing. Their activity is further modulated by neurotransmitters, including serotonin and norepinephrine, which provide flexibility in sensorimotor integration. Pathological conditions such as chronic stress and sodium channel dysfunction induce MTN hyperexcitability or irregular firing, contributing to bruxism, temporomandibular disorders, and feeding impairment in amyotrophic lateral sclerosis models. In addition, aging and tooth loss lead to Piezo2 downregulation and neuronal death, potentially resulting in masticatory dysfunction and cognitive decline. Recent findings suggest that interventions targeting vesicular glutamate transporter 1 projections, melanocortin 4 receptor signaling, and nitric oxide pathways represent novel therapeutic approaches. Taken together, MTNs have emerged as promising targets for treating conditions ranging from masticatory motor disorders to neurodegenerative diseases.

## Graphical abstract

**Figure:**
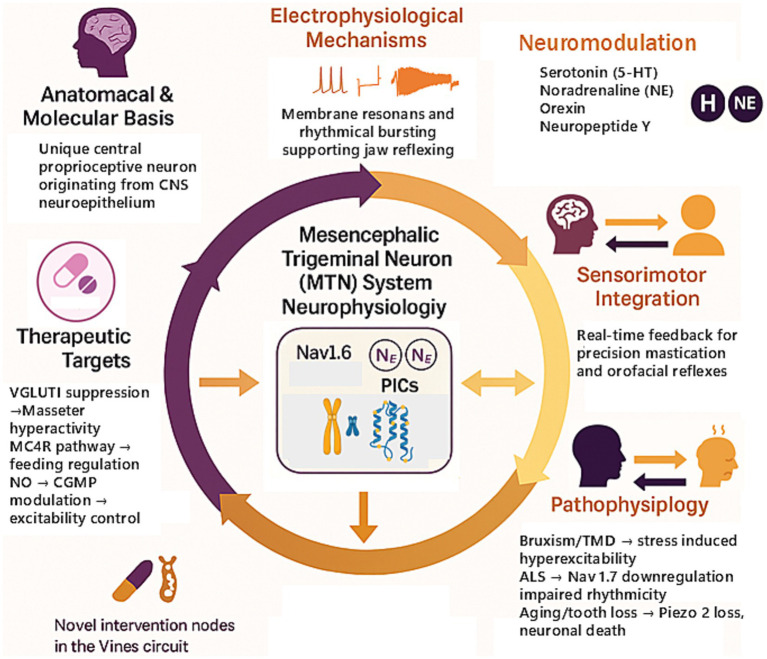
Graphical abstract

## 1 Introduction

Orofacial movements such as speaking, eating, and expressing emotions are executed unconsciously under remarkably precise control. At the foundation of this fine motor regulation lies proprioception, the sensory system that provides continuous feedback regarding the position, movement, and forces acting on the body. In mastication, during which millisecond-level precision in force adjustment is required, proprioceptive input is indispensable. Mesencephalic trigeminal neurons (MTNs) represent the central nodes of orofacial proprioception (Lazarov, 2002). MTNs are distinguished by their exceptional anatomical configuration, which underscores their role as “guardians” in maintaining orofacial function.

The most striking feature of MTNs is their highly unusual localization: unlike most first-order sensory neurons, whose cell bodies reside in peripheral sensory ganglia (e.g., the trigeminal ganglion), MTNs are located within the midbrain of the central nervous system (CNS; Ramón y Cajal, 1909). This exceptional arrangement suggests that MTNs serve as both conduits of sensory information and active participants in higher-order information processing and rapid reflex control. Their peripheral processes terminate in mechanoreceptors within the muscle spindles of jaw-closing muscles and in the periodontal ligament, which anchors the teeth to alveolar bone (Shigenaga et al., 1990; Linden et al., 1994). Whereas muscle spindles monitor muscle stretch, periodontal ligament receptors detect pressure and vibration applied to the teeth. MTNs integrate these two distinct sensory streams in real time, providing precise information on the state of the masticatory apparatus and occlusal forces (Kato et al., 1999).

This unique capacity for high-fidelity information gathering underpins the concept of MTNs as “guardians” of oral function. For example, when chewing hard food, humans can crush it efficiently without damaging their teeth or temporomandibular joint because MTNs rapidly feedback occlusal force signals from the periodontal ligament to the trigeminal motor nucleus, optimizing the contractile force of jaw-closing muscles (Morimoto et al., 1985, 1989). Excessive force elicits inhibitory input from MTNs to jaw-closing muscles, whereas insufficient force enhances contraction. Through this finely tuned feedback loop, teeth, periodontal tissues, and temporomandibular joints are protected from mechanical overload. MTNs also receive input from jaw-closing muscle spindles and serve as the afferent limb of the monosynaptic jaw-jerk reflex (Szentágothai, 1948). This rapid defensive reflex contracts jaw-closing muscles within milliseconds when the mandible is unexpectedly depressed, maintaining joint stability. The central role of MTNs in this reflex arc provides compelling evidence of their indispensable contribution to orofacial homeostasis.

Pathological consequences can arise when this protective function is compromised. Abnormally strong masticatory muscle activity observed in sleep bruxism has been linked to dysfunction of proprioceptive feedback mediated by MTNs (Giovanni and Giorgia, 2021). Similarly, in atypical odontalgia and certain forms of trigeminal neuralgia, aberrant processing of proprioceptive input has been hypothesized to contribute to altered pain perception, potentially underpinned by abnormal electrophysiological properties of MTNs (Sessle, 2006).

Taken together, MTNs, through their unique anatomical localization and functional connectivity, enable fine control of mastication, provide reflexive protection of orofacial structures, and contribute to the oral representation of “self-awareness.” These neurons are active guardians of orofacial integrity. Several review articles on MTNs have been published to date (Table 1). This review focuses on the electrophysiological properties of MTNs, highlighting the mechanisms underlying their precise functional roles and exploring the contributions of dysfunction to several orofacial disorders.

**Table 1:** Previous review articles on MTNs (Vmes/MTN).

Author(s), year	Journal	Main focus	Key contributions	Limitations/gaps
Shigenaga et al. (1988)	*Brain Research Reviews*	Morphology and connectivity	Detailed anatomical mapping of MTN projections	Limited to cat morphology; small sample, little functional or developmental data
Sakata and Yoshimatsu (1995)	*Methods and Findings in Experimental and Clinical Pharmacology*	Role of hypothalamic and Vmes histamine neurons in energy balance and feeding	Found that histamine suppresses feeding, regulates mastication (Vmes), controls meal size (VMH), and links energy deficit to astrocytic glycogenolysis; deficits explain obesity in Zucker rats	Mostly animal models (rats); limited direct human evidence
Lazarov (2000)	*Advances in Anatomy, Embryology and Cell Biology*	Neuroanatomy and neurochemistry of cat MTN	Identified Glu as primary transmitter; presence of small GABAergic neurons, NOS, and peptidergic inputs; evidence of plastic changes after axotomy	Species specificity unclear; limited functional validation; many findings based on abnormal conditions
Lazarov (2002)	*Progress in Neurobiology*	Comparison of neurochemical content of TG vs. MTN	Identified distinct neurotransmitter and peptide profiles in TG and MTN neurons; observed Glu, PV, and CB dominance in MTN vs. diverse neuropeptides in TG; highlighted unique perineuronal basket-like arborizations	Lacks functional analysis of how these neurochemical differences influence sensory processing; based mostly on immunohistochemistry without physiological correlation
Sakata et al. (2003)	*Experimental Biology and Medicine*	Role of MTN histamine (HA) neurons in mastication and energy regulation	Found that HA neurons in Vmes are activated at early feeding phase; the neurons regulate eating speed and link mastication to hypothalamic centers controlling intake and metabolism	Study limited to rats; mechanisms in humans remain unclear; exact molecular pathways beyond HA remain unresolved
Kolta et al. (2007)	*Archives of Oral Biology*	Burst generation in trigeminal neurons	Revealed these neurons generate rhythmic bursts independent of synaptic input; maturation coincides with onset of mastication; low extracellular Ca^2+^ helps initiate chewing	Study limited to rats; developmental stage only; no direct behavioral or human validation
Lazarov (2007)	*Brain Research Reviews*	Neurochemistry of MTN in orofacial proprioception	Summarizes neurotransmitters, neuropeptides, receptors in MTN; highlights role in masticatory proprioception; provides evidence for neuroplasticity depending on environment	Mostly descriptive review; limited functional/physiological data; lacks direct experimental validation of proposed mechanisms
Xing et al. (2014)	*Neurosignals*	Electrophysiological features	Systematic overview of MTN firing properties	Limited molecular/pathophysiological sequencing
Yoshida et al. (2017)	*Journal of Oral Science*	Projection and synaptic organization of single Vmes afferents	Compared spindle and periodontal afferents; revealed distinct projection and synaptic patterns in jaw-closing and supratrigeminal nuclei	Lack of detailed prior reports; limited to single-afferent level
Trigo et al. (2025)	*Neuroscience*	Role of electrical coupling and Ih current in coincidence detection in MesV neurons	Ih makes coupling time-dependent, sharpens coincidence detection of inhibitory inputs	Model-based, limited *in vivo* evidence

## 2 Anatomical organization

### 2.1 Location, connections, and projections of the mesencephalic trigeminal nucleus (Vmes)

**Figure 1:**
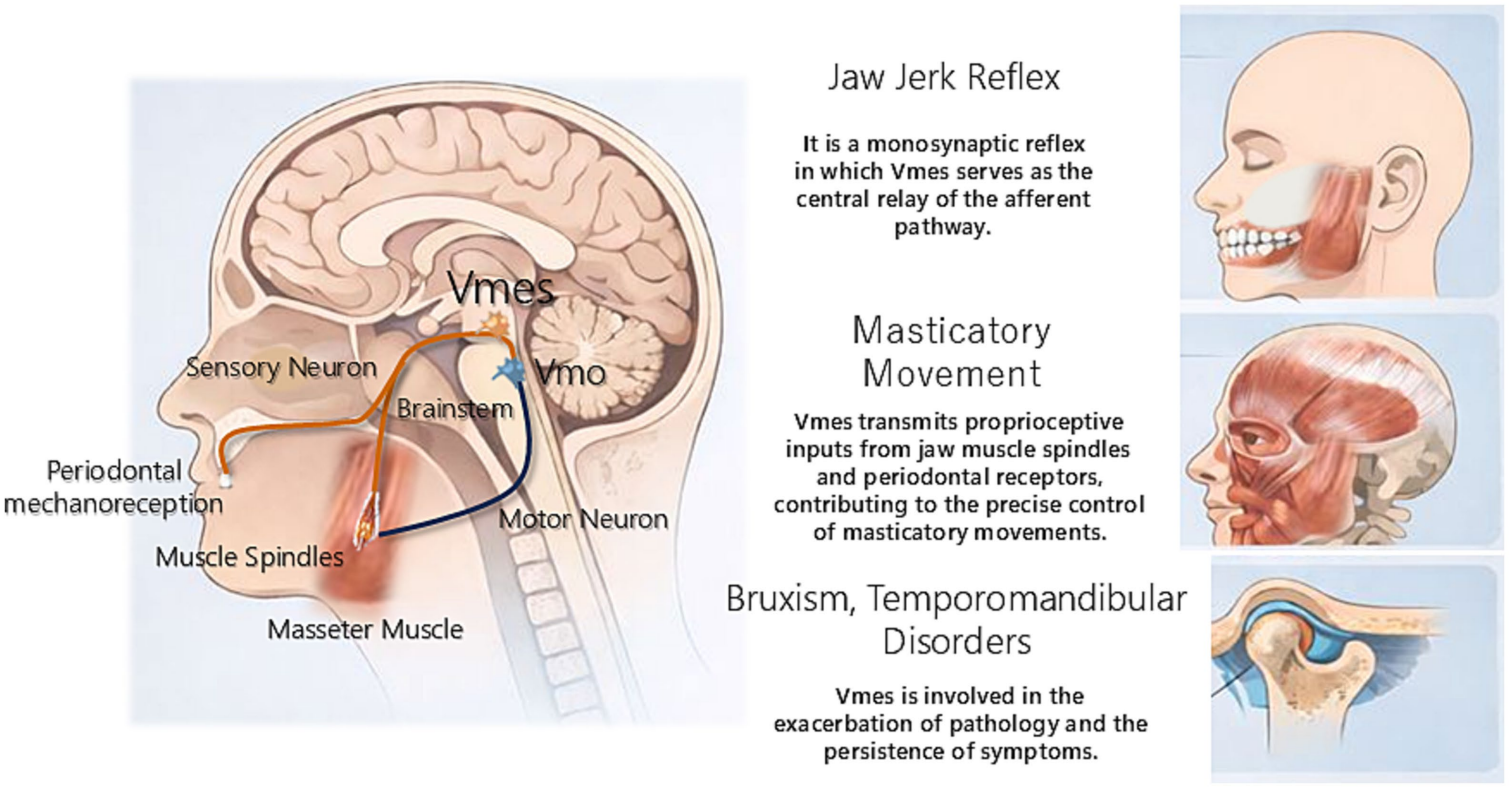
Anatomical localization and projection targets of the mesencephalic trigeminal nucleus. Schematic representation of the anatomical localization of Vmes and its major peripheral and central connections. Neurons within the Vmes receive proprioceptive input from muscle spindles of jaw-closing muscles and mechanoreceptors in the periodontal ligament. Central projections from the Vmes form monosynaptic connections with Vmo, constituting the afferent limb of the jaw-jerk reflex and supporting precise control of masticatory movements.

The Vmes contains the cell bodies of unique primary sensory neurons mediating orofacial proprioception. Unlike conventional sensory neurons located in peripheral ganglia such as the trigeminal ganglion (TG), the cell bodies of MTNs reside within the brainstem, a central localization that reflects their specialized function (Lazarov, 2002). The Vmes forms a longitudinal column extending from the level of the superior colliculus, sometimes reaching the pretectal area and interstitial nucleus of Cajal, caudally surrounding the cerebral aqueduct and fourth ventricle through the inferior colliculus and locus coeruleus. The Vmes extends down to the pontine level adjacent to the principal sensory trigeminal nucleus and dorsomedial trigeminal motor nucleus (Vmo; Huff et al., 2025). This elongated organization corresponds to the developmental origin of MTNs from the rhombic lip rather than the neural crest (Shirasaki and Pfaff, 2002). Functionally, neurons innervating jaw-closing muscles (masseter, temporalis) occupy rostral and central portions, whereas those projecting to jaw-opening muscles and the periodontal ligament are located caudally (Luo and Dessem, 1996) (Figure 1).

Mesencephalic trigeminal neurons are pseudounipolar neurons whose single process bifurcates into peripheral and central branches. The peripheral branch joins the trigeminal motor root (mandibular nerve) and terminates in two major proprioceptor types: muscle spindles in jaw-closing muscles, sensing stretch (Kato et al., 1999), and mechanoreceptors in the periodontal ligament, detecting tooth pressure (Matsushita et al., 1982). Through these proprioceptors, the Vmes provides continuous information on jaw movement and occlusal force.

The central branches of MTNs project widely within the brainstem. The most prominent branch is the direct excitatory projection to the Vmo, forming the monosynaptic jaw-jerk reflex arc that elicits jaw-closing muscle contraction upon stretch (Appenteng et al., 1978; Chandler, 1989). Additional collaterals target orofacial motor and autonomic nuclei, including the trigeminal sensory nuclei, reticular formation, and superior salivatory and hypoglossal nuclei (Lazarov, 2002). In rats, jaw muscle spindle afferents can drive multiple orofacial motoneuron pools via common premotor neurons (Zhang et al., 2012), indicating that Vmes input coordinates complex multi-muscle movements such as jaw–tongue–facial interactions. Some projections also reach the cerebellum (especially the anterior lobe), contributing to motor coordination and learning, and possibly extend polysynaptically to the thalamus for proprioceptive awareness (Lazarov, 2000).

Through this unique central localization and extensive projection network, the Vmes serves as a crucial hub integrating proprioceptive input for reflexive and coordinated orofacial motor control.

### 2.2 Developmental origins and molecular markers

Although migratory neural crest cells give rise to nearly all primary sensory neurons located in peripheral ganglia, MTNs arise directly from the neuroepithelium of the CNS. This unique developmental trajectory is intrinsically linked to a distinctive combination of molecular markers that govern their fate, survival, and eventual specialization in proprioceptive function.

According to the classical paradigm of neurodevelopment, the central nervous system originates from the neural tube, whereas the peripheral nervous system primarily derives from the neural crest, reflecting distinct developmental lineages. Based on this framework, early studies often assumed MTNs, like TG neurons, were neural crest-derived (D'Amico-Martel and Noden, 1983). However, subsequent evidence from avian chimera experiments and lineage-tracing studies has clearly refuted this assumption, demonstrating that MTNs arise through a distinct ontogenetic pathway.

Current consensus indicates that the majority of MTNs originate from the dorsal neuroepithelium at the midbrain–hindbrain junction, encompassing the isthmic organizer region and the most rostral rhombomere (Hunter et al., 2001). This dorsal neuroepithelial domain includes the rhombic lip, a secondary germinal zone known to generate multiple neuronal populations, including cerebellar granule cells.

Lineage-tracing studies using Cre recombinase systems driven by promoters active within this neuroepithelial territory, such as Wnt1 and Engrailed-1, have provided compelling evidence that progenitor cells expressing these transcription factors migrate from the ventricular zone and differentiate into MTNs (Danielian et al., 1998; Zervas et al., 2004). The shared developmental origin of MTNs and certain cerebellar neurons, under the influence of potent signaling molecules such as Wnt1 and Fgf8 secreted from the isthmic organizer, suggests the presence of a common developmental program underlying neuronal populations involved in sensorimotor integration.

Notably, neurons derived from this isthmic organizer–associated neuroepithelium, including cerebellar granule cells, share electrophysiological properties optimized for high temporal precision, such as reliable high-frequency firing and subthreshold resonance. Consistent with this, MTNs exhibit specialized intrinsic membrane properties that support precise timing of proprioceptive signaling, reinforcing the functional relevance of their shared developmental origin.

The specification of progenitors into mature MTNs is tightly regulated by the spatiotemporal expression of transcription factors. This process likely begins with the expression of the paired-box transcription factors Pax3 and Pax7 in the dorsal neural tube (Baker et al., 1999). Once these cells exit their final mitosis and commit to the MTN lineage, they begin to express a distinctive set of transcription factors that promote their differentiation from other rhombic lip-derived neurons. The most critical of these factors are the paired-like homeodomain transcription factors Phox2a and Phox2b, known master regulators of visceral sensory and motor neuron development that are believed to contribute to MTN identity and survival (Pattyn et al., 2000). At the same time, MTNs express the LIM homeodomain protein Islet-1 and the POU domain transcription factor Brn3a (also known as Pou4f1), both broadly involved in sensory neuron differentiation and axon guidance (Fedtsova and Turner, 1995).

The establishment of MTNs as proprioceptive neurons is marked by the expression of Runt-related transcription factor 3. Runt-related transcription factor 3 defines proprioceptive neurons across the nervous system, including dorsal root ganglion (DRG) neurons, and its expression in MTNs highlights a molecular homology with peripheral proprioceptive neurons despite their distinct locations and origins (Inoue et al., 2002). As neurons mature, their survival and circuit integration increasingly depend on trophic support. Although many DRG proprioceptive neurons rely on neurotrophin-3 acting through TrkC receptors, MTNs express TrkB, implicating BDNF–TrkB signaling in their maintenance and function (Tang et al., 2010). This divergence in neurotrophic dependence underscores a fundamental distinction between central and peripheral proprioceptive systems.

### 2.3 Piezo2 as a molecular basis of mechanotransduction in MTNs

Mechanotransduction is the process by which mechanical stimuli are converted into electrical signals in sensory neurons. Piezo2, a mechanically activated cation channel, has been identified as the principal molecular mediator of mechanotransduction in mammalian proprioceptive neurons. Genetic ablation of Piezo2 in mice abolishes mechanically evoked receptor potentials and action potential firing in muscle spindle afferents, resulting in profound deficits in proprioceptive signaling and severe motor incoordination. These findings establish Piezo2 as an essential mechanotransducer underlying peripheral proprioception (Woo et al., 2015).

Mesencephalic trigeminal neurons exhibit functional and molecular characteristics closely resembling those of peripheral proprioceptive neurons, despite their unique localization within the central nervous system (Lazarov, 2002). MTNs innervate muscle spindles of jaw-closing muscles and mechanoreceptors in the periodontal ligament, thereby encoding muscle stretch and tooth load information critical for the precise control of mastication (Linden et al., 1994). Consistent with their proprioceptive identity, MTNs express Piezo2, providing a molecular substrate for the conversion of mechanical deformation into receptor potentials at their peripheral terminals (Florez-Paz et al., 2016).

Piezo2-mediated mechanotransduction is well suited to the physiological demands placed on MTNs. The fast activation kinetics and low mechanical threshold of Piezo2 channels enable rapid and faithful detection of subtle changes in muscle tension and occlusal force, analogous to the requirements of limb proprioceptive systems. Mechanical inputs transduced via Piezo2 are conveyed centrally to brainstem circuits, including direct projections to the trigeminal motor nucleus, thereby forming the afferent limb of the jaw-jerk reflex and contributing to fine-tuned regulation of bite force and masticatory rhythm (Linden et al., 1994; Lazarov, 2002; Woo et al., 2015; Florez-Paz et al., 2016).

Collectively, Piezo2 endows MTNs with high-fidelity mechanosensory capability, linking peripheral mechanical events in muscle spindles and periodontal ligament receptors to central sensorimotor integration. Through this mechanism, Piezo2 serves as a key molecular gatekeeper supporting the precise timing and force control required for normal mastication (Linden et al., 1994; Lazarov, 2002; Woo et al., 2015; Florez-Paz et al., 2016).

### 2.4 Morphological and neurochemical diversity within the Vmes population

MTNs are characterized by an unusually high degree of electrical coupling. Their morphology resembles, yet diverges from, that of peripheral sensory ganglia (Shigenaga et al., 1988; Ter-Avetisyan et al., 2018). Intracellular horseradish peroxidase tracing in cats revealed the distinctive “Y-shaped” bifurcation of Vmes axons, consisting of a peripheral branch projecting to cranial nerves and a central branch innervating brainstem motor and reticular nuclei (Shigenaga et al., 1990). These central branches exhibit axon collaterals terminating in the dorsal Vmo or trigeminal-adjacent structures, with collateral patterns correlating to group I (muscle spindle) or group II (periodontal) sensory phenotypes (Shigenaga et al., 1990). Developmental studies further indicated that these morphological features are established early. In mice, molecular pathways such as CNP–Npr2–cGKI signaling are essential for proper axonal bifurcation at the hindbrain level, highlighting canonical growth patterns that diversify morphology within the Vmes pool (Ter-Avetisyan et al., 2018).

Beyond morphology, the Vmes displays a rich neurochemical repertoire. In primates, ultrastructural studies combining immunoelectron microscopy demonstrated that although the somata are sparsely innervated, they receive diverse synaptic inputs, including excitatory and modulatory signals (Wang and May, 2023). In rodents, immunohistochemical surveys have revealed the presence of multiple neurotransmitters, including glutamate (via VGLUT1/2), GABA, serotonin (5-HT), and neuropeptides such as dynorphin A and orexin. MTNs also exhibit ATP-gated P2X receptor currents, implicating purinergic input in excitatory control (Khakh et al., 1997). Moreover, during early postnatal development, trigeminal neurons display differential expression of the NMDA receptor subunits NR2A and NR2B, with a developmental shift from NR2B to NR2A dominance that dictates excitatory postsynaptic current kinetics and synaptic plasticity rules (Turman et al., 1999; Turman et al., 2002; Turman et al., 2000).

Electrophysiologically, this molecular heterogeneity translates into functional diversity. Recordings in the cat Vmes uncovered differences between somatodendritic spikes and axonal action potentials, as well as variable responses to mechanical stretch (Shigenaga et al., 1990). Recent single-cell transcriptomic analyses in mice revealed at least five transcriptionally distinct Vmes subtypes, each expressing different combinations of ion channels, receptors, and mechanosensory proteins. These subtypes likely underlie group-specific responses to muscle versus ligament stretch (Lee et al., 2023).

Thus, the morphological and neurochemical diversity of the Vmes reflects its dual roles as both a primary sensor and integral component of premotor circuits. Its large bifurcating axons enable efficient transmission of proprioceptive signals to jaw motor nuclei while supporting local electrical propagation. Simultaneously, the broad range of synaptic inputs—from glutamatergic to peptidergic—provides dynamic modulation under different behavioral and stress conditions. The diversity within the Vmes neuronal population therefore establishes a neuroanatomical framework that supports both reflexive jaw control and adaptive plasticity.

## 3 The electrophysiological repertoire of MTNs

### 3.1 Intrinsic properties: components of excitability

Mesencephalic trigeminal neurons are unique primary sensory neurons within the mammalian CNS, as their cell bodies are located inside the CNS and they transmit proprioceptive information from the muscle spindles of the masticatory muscles and the periodontal ligament (Yoshida et al., 2017). The electrophysiological excitability of these neurons is largely dependent on their intrinsic properties, particularly the expression patterns and distribution of Na^+^ and K^+^ channels, which play a crucial role in determining firing modes. MTNs exhibit intrinsically generated, depolarization-induced high-frequency spike discharges that are repeatedly and intermittently evoked during a depolarizing current pulse, rather than occurring spontaneously (Wu et al., 2001). This firing behavior is supported by membrane potential resonance arising from the interaction between persistent Na^+^ currents and 4-AP-sensitive non-inactivating (D-type; I_D) K^+^ currents, with additional contributions from other K^+^ conductances shaping excitability (Wu et al., 2001; Wu N. et al., 2005; Wu W. W. et al., 2005; Enomoto et al., 2006). Notably, glutamatergic synaptic activation (including mGluR-I–dependent mechanisms) can further enhance resonance-dependent oscillations and promote a switch from single spiking to bursting in MTNs (Chung et al., 2015). Furthermore, researchers demonstrated that these neurons generate persistent inward currents (PICs), which enable them to sustain firing over extended periods (Enomoto et al., 2006). These intrinsic properties are also known to change plastically during development or under pathological conditions, suggesting that MTN dysfunction can contribute to abnormal mastication or altered nociception (Turman et al., 1999). Collectively, these findings underscore the importance of intrinsic excitability in supporting the sensorimotor integration functions of MTNs.

Mesencephalic trigeminal neurons also exhibit different firing patterns than other sensory neurons. These neurons process proprioceptive input from muscle spindles and display both tonic firing and rhythmic bursting. According to prior research, MTNs can sustain stable repetitive action potential discharge, which might vary across developmental stages (Yoshida et al., 2017). Shigenaga et al. (1988), further demonstrated that during rhythmic mastication, MTNs burst in synchrony with jaw motor neurons, suggesting functional coupling with central pattern generators. These diverse firing patterns indicate that MTNs are endowed with adaptive response properties required for fine control of both static and dynamic muscle tension.

**Figure 2:**
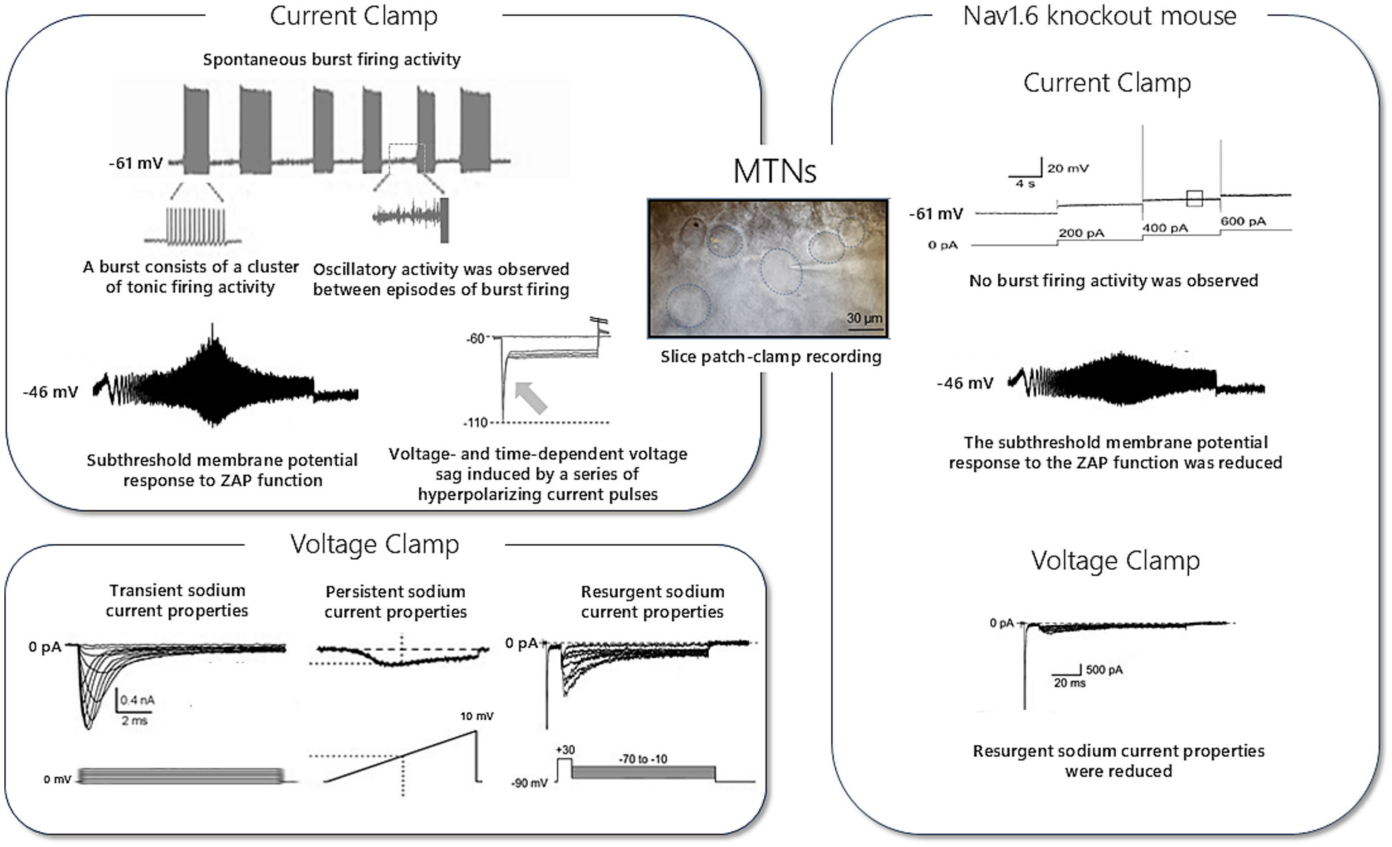
Electrophysiological properties of mesencephalic trigeminal neurons. Representative electrophysiological properties of mesencephalic trigeminal neurons recorded by slice patch clamp. Wild-type neurons exhibit spontaneous burst firing, subthreshold membrane potential oscillations, voltage sag responses, and membrane resonance revealed by ZAP stimulation. Voltage-clamp recordings demonstrate transient, persistent, and resurgent sodium currents. In Nav1.6 knockout mice, burst firing and subthreshold resonance are absent, and resurgent sodium currents are markedly reduced, indicating a critical role of Nav1.6 in rhythmic firing.

In addition to spontaneous activity, MTNs sometimes display subthreshold membrane potential oscillations, characterized by small, periodic fluctuations within a given voltage range that can lead to action potential generation under appropriate conditions. This phenomenon, observed *in vitro* by Pedroarena and Llinás (1997), was identified as an important electrical signature suggestive of pacemaker-like properties. Specifically, low-threshold Ca^2+^ currents (T-type Ca^2+^ currents) and persistent Na^+^ currents help stabilize these oscillations and form the basis for rhythmic activity (Enomoto et al., 2006). Moreover, the presence of hyperpolarization-activated (I_h_) currents in MTNs, contributing to phase and frequency alignment of subthreshold oscillations via hyperpolarization sag and rebound depolarization, has long been recognized (Khakh and Henderson, 1998). This suggests that MTNs participate in rhythm generation, thereby contributing to the timing of cyclic movements such as mastication. Furthermore, MTNs receive synaptic inputs that evoke firing, reset the phase, and modulate the frequency and amplitude of intrinsic oscillations (Verdier et al., 2004). Thus, pacemaker-like activity in the Vmes functions as a “semi-autonomous oscillator” entrainable by network inputs and tightly linked to synaptic integration (Figure 2).

### 3.2 Ionic basis of excitability: major channels and currents

The excitability of MTNs is maintained through the coordinated action of multiple ion channels regulating membrane potential. The most critical are voltage-dependent sodium (Nav) channels, which generate action potentials with rapid upstrokes. Yoshida demonstrated that Vmes neurons regulate excitability through Na^+^ and K^+^ currents as well as Ca^2+^ currents (Yoshida et al., 2017). Furthermore, Del Negro and Chandler revealed that PICs are central to sustaining excitability in the Vmes, with Na^+^-PICs and Ca^2+^-PICs forming the basis for rhythmic firing (Enomoto et al., 2006). Inhibitory channels such as inwardly rectifying K^+^ channels and delayed rectifier K^+^ channels are also essential for setting firing thresholds and recovery dynamics, thereby refining motor control and preventing malfunction. Additionally, Yamada and colleagues, using neonatal rats, demonstrated that zinc deficiency alters MTN membrane properties and spike discharge patterns. Specifically, zinc-deficient neurons exhibited moderate membrane potential depolarization, reduced cell capacitance, increased afterhyperpolarization, shortened burst duration, and accelerated spike frequency adaptation. These effects were likely attributable to enhanced Ca^2+^-dependent K^+^ conductance, suggesting that the nutritional status contributes to the plasticity of firing modes (Yamada et al., 2021).

In MTNs, the interplay among voltage-dependent Na^+^, K^+^, and Ca^2+^ channels is crucial for action potential generation and firing pattern regulation. Notably, Nav1.6 channels mediate transient, persistent (I_NaP_), and resurgent sodium (I_NaR_) currents, making major contributions to firing frequency and membrane resonance properties, as demonstrated in knockout mouse studies (Enomoto et al., 2006; Seki et al., 2019). Deletion of Nav1.6 resulted in an 18% reduction in transient Na^+^ currents, 39% reduction in I_NaP_ currents, and 76% reduction in I_NaR_ currents, leading to marked changes in membrane resonance (Enomoto et al., 2006; Westenbroek et al., 1989). Enomoto and colleagues further demonstrated that developmental increases in I_NaP_ and I_NaR_ currents are critical for supporting high-frequency firing, providing a basis for age-dependent changes in MTN excitability (Enomoto et al., 2018; Venugopal et al., 2019).

Voltage-dependent calcium channels, particularly T-type channels, are also implicated in subthreshold oscillations and burst firing. K^+^ channels, including delayed rectifier K^+^ channels and inwardly rectifying K^+^ channels, contribute to repolarization and recovery, regulating firing timing and patterns (Tanaka et al., 2003). In neonatal rat MTNs, delayed rectifier and A-type K^+^ currents were found via both physiological recordings and computational models to primarily regulate the onset, frequency, and adaptation of repetitive firing (Del Negro and Chandler, 1997). In this context, Tanaka and colleagues reported functional maturation of inwardly rectifying K^+^ channels and I_h_ currents during development, with increased I_h_ currents providing low-frequency resonance and firing stability (Tanaka et al., 2003). Together, these channels establish the ionic basis that supports stable excitability and rhythmic activity in the Vmes.

The pacemaker function of MTN is sustained by I_NaP_ currents and I_h_ currents mediated by HCN channels (Khakh and Henderson, 1998). In rat MTNs, I_h_ currents have been physiologically identified as Cs^+^-sensitive currents activated by hyperpolarization, providing the ionic substrate for sag responses and rebound firing (Khakh and Henderson, 1998). Prior research indicated that I_NaP_ currents contribute to burst firing and resonance by facilitating subthreshold oscillations, thereby enabling rhythmic activity (Wu et al., 2005; Enomoto et al., 2006). I_h_ currents, mediated by HCN channels (particularly HCN1 and HCN2), are activated at subthreshold voltages, and they contribute to membrane stabilization and spike timing regulation (Wu et al., 2005). Kang et al. showed that I_h_ and Na^+^–K^+^ pump currents in MTNs interact bidirectionally via local Na^+^ microdomains: Na^+^ influx through h-channels activates pump currents, while pump activity modulates I_h_. Colocalization of HCN1/2 channels and the Na^+^–K^+^ pumpalpha3 isoform supports this reciprocal regulation of neuronal excitability (Kang et al., 2004). Because this Na^+^ microdomain is also shared with AMPA receptors, it was concluded that I-AMPA evoked in MTN neurons is opposed by a transient decrease in standing inward Ih due to negative shifts in the reversal potential of I_h_ as a consequence of elevated Na^+^ concentrations within the Na^+^ microdomain in response to Na^+^ influx through AMPA channels (Kawasaki et al., 2018). Accordingly, suppression of HCN conductance-for example by Cs^+^ application or noradrenergic inputs from the locus coeruleus-enhances AMPA-evoked depolarization and can trigger a barrage of spikes in MTNs (Kawasaki et al., 2018; Toyoda et al., 2022). This mechanism is distinct from the previously postulated mechanisms for the inhibition of EPSPs by the activity of HCN channels (Magee, 1998; Carr et al., 2007). Tanaka and colleagues further reported that 5-HT suppresses I_h_ currents, particularly in late developmental stages, suggesting developmental stage-dependent serotonergic modulation of MTN frequency selectivity (Tanaka et al., 2019). This dynamic interaction tunes sag responses, rebound spikes, resonance frequency, and spike precision, thereby stabilizing tonic firing and enhancing the robustness of rhythmogenesis under sustained input (Tanaka et al., 2019). Pharmacological manipulation of HCN activity or Na^+^–K^+^ pump activity shifts these parameters in predictable ways, supporting the causal role of this bidirectional interaction. Tanaka and colleagues also reported that orexin A enhances I_NaP_ currents, increasing firing frequency and resonance, thereby suggesting a link among feeding behavior, energy metabolism, and orofacial motor control (Tanaka et al., 2021) (Figure 2).

### 3.3 Synaptic integration and neuromodulation

Mesencephalic trigeminal neurons, owing to their unique structure and function, are subject to both sensory input reception and integration and regulation by a wide range of neurotransmitters. Copray and coworkers immunohistochemically demonstrated the expression of receptors for 5-HT, norepinephrine (NE), dopamine, and GABA in MTNs (Copray et al., 1990). These neuromodulators can alter resting membrane potential, firing threshold, and postsynaptic potentials, thereby flexibly adjusting neuronal excitability. Synaptic integration and neuromodulation by extrinsic inputs modify MTN activity patterns and timing, enabling dynamic regulation of cyclic movements and reflexes. Purinergic modulation by ATP also plays a critical role. In rat MTNs, “native” P2X receptors mediate fast inward currents evoked by ATP, but their pharmacological and kinetic profiles do not correspond to any of the known P2X subtypes (P2X1–P2X7) characterized in heterologous systems (Khakh et al., 1997; Patel et al., 2001). Moreover, MTN afferents converge and diverge within the premotor layer, distributing in parallel to multiple motor pools via “common premotor neurons.” Thus, synaptic integration both regulates gain within a single motor nucleus and organizes coordination across muscle groups (Zhang et al., 2012). This “mismatch” suggests that P2X receptors functioning in MTNs possess unique subunit compositions or accessory factor dependencies, indicating the existence of MTN-specific purinergic regulatory mechanisms.

Autapses, self-synapses in which a neuron provides input to itself, have also been hypothesized in MTNs. Using computational modeling, Guo and colleagues demonstrated that autaptic self-regulation can contribute to firing stability and timing precision (Guo et al., 2016). If MTNs employ such a self-regulatory mechanism, it might function as an intrinsic corrective system for fine-tuning masticatory rhythms and sensorimotor integration.

MTNs are strongly influenced by extrinsic neuromodulators, particularly 5-HT and NE. 5-HT depolarizes MTNs and lowers their firing threshold, thereby increasing excitability (Hsiao et al., 1997; Tanaka and Chandler, 2006). Tanaka and colleagues also demonstrated that 5-HT suppresses I_h_ currents and alters low-frequency resonance, indicating that serotonergic modulation developmentally shifts frequency selectivity (Tanaka et al., 2019). Noradrenergic inputs from the locus coeruleus suppress I_h_ conductance in MTNs via activation of α2-adrenergic receptors, likely through volume transmission mechanisms (Toyoda et al., 2022). This suppression of I_h_ alters intrinsic excitability and can facilitate depolarization-induced spike barrages in response to glutamatergic inputs, consistent with the dual functional roles of MTNs as primary sensory relays and premotor-like elements within trigeminal sensorimotor circuits (Kang et al., 2004; Chung et al., 2015; Kawasaki et al., 2018; Toyoda et al., 2022; Kang et al., 2024). These modulatory mechanisms are essential for adjusting MTN function in response to environmental stimuli and emotional states. Moreover, ATP released from peripheral tissues such as periodontal ligament and muscle spindles during mechanical stimulation can provide rapid excitatory input to MTNs via native P2X receptors (Khakh et al., 1997; Tanaka and Chandler, 2006). Of particular note, the pharmacological profile of P2X-mediated currents recorded in rat MTNs does not match those of known heterologously expressed P2X receptors (Khakh et al., 1997; Patel et al., 2001). This finding suggests MTN-specific P2X receptor subtypes or accessory protein dependence, prompting reconsideration of ATP signaling in fine-tuning reflex gain and firing timing. From a therapeutic perspective, although generalized P2X antagonists might have unpredictable efficacy, identifying MTN-specific purinergic pathways could open opportunities for selective intervention.

Furthermore, Tanaka and colleagues reported that orexin A, a hypothalamic neuropeptide involved in feeding behavior and energy metabolism, acts on MTNs to enhance I_NaP_ currents, thereby increasing firing frequency and resonance. This indicates that the rhythmic control of oral movements can be modulated by hypothalamic peptides, revealing a novel link between the neural basis of mastication/swallowing and systemic metabolic regulation (Tanaka et al., 2021). Additionally, Seki and colleagues reported that neuropeptide Y (NPY), a potent orexigenic peptide, directly acts on MTNs. NPY administration induced membrane depolarization and inward currents, shortened afterhyperpolarization, and increased spike frequency and burst rates. These effects were mediated primarily by Y1 and Y5 receptors, suggesting that NPY modulates feeding behavior, orofacial reflexes, and proprioceptive control of mastication (Seki et al., 2020) (Table 2).

**Table 2:** Electrophysiological properties of MTNs, listing major ion channels and currents identified to date.

Channel/current	Electrophysiological feature	Experimental approach	Functional role	Key references
Nav1.6-dependent Na^+^ current	Sustained repetitive firing; reduced transient, persistent, resurgent I in KO mice	Whole-cell recording from Nav1.6-null mice	Critical for burst generation and resonance	Enomoto et al. (2007)
I_h_ currents	Pacemaker-like activity	Intracellular recordings	Regulates timing and rhythmicity	Wu et al. (2001)
Persistent Na^+^ current	Supports rhythmic bursting	Whole-cell patch	Maintains excitability and sensory responsiveness	Enomoto et al. (2006)
Piezo2 channel	Mechanotransduction	Molecular expression studies	Mediates proprioceptive input from jaw muscles	Woo et al. (2015)
Low-threshold Na^+^ + delayed rectifier K^+^	Sustained spontaneous firing; sharp threshold via coordinated Na^+^ and K^+^ currents	Whole-cell patch in rat MTN; pharmacology (TTX, 4-AP)	Shapes excitability/threshold; supports tonic and burst output	Del Negro and Chandler (1997), Wu et al. (2005), and Saito et al., 2006
PICs (Na^+^-PIC, Ca^2+^-PIC)	Long-lasting repetitive firing; enhanced membrane resonance	Voltage-clamp and computational modeling	Maintains excitability and rhythmic bursting over prolonged inputs	Enomoto et al. (2006)
Subthreshold oscillations (Na^+^, T-type Ca^2+^)	Periodic small-amplitude Vm oscillations below threshold	In vitro slice recordings	Pacemaker-like activity; burst initiation and timing	Pedroarena and Llinás (1997) and Enomoto et al. (2006)
HCN-mediated I_h_ currents (HCN1/HCN2)	Hyperpolarization sag; rebound depolarization; phase control	Whole-cell patch and pharmacology (Cs/ZD7288)	Stabilizes Vm and controls timing and rhythmicity	Khakh and Henderson (1998)
Na^+^–K^+^ pump × I_h_ interaction	Bidirectional I_h_–Na^+^–K^+^ pump interactions via Na^+^ microdomains	Whole-cell patch and pharmacological manipulation of HCN and Na^+^–K^+^ pump	Regulation of resting membrane potential and neuronal excitability	Kang et al. (2004)
Zinc deficiency effect (Ca^2+^-dependent K^+^ conductance)	Depolarized Vm; reduced capacitance; larger AHP; shortened burst duration; accelerated spike frequency adaptation	Whole-cell patch in neonatal rat MTN (ZD vs. control diet)	Nutritional state alters excitability via increased Ca^2+^-dependent K + conductance	Yamada et al. (2021)
I_NaR_/I_NaP_ developmental increase	Quantitative increase of resurgent and persistent Na^+^ currents with age	Whole-cell patch clamp across developmental stages	Supports age-dependent high-frequency firing in MTN	Enomoto et al. (2018)
Kir/I_h_ developmental maturation	Increase of I_h_ currents during P2-12; enhanced low-frequency resonance	Voltage-clamp in developing rat MTN	Stabilizes resting potential and firing via inward rectification and I_h_ currents	Tanaka et al. (2002)
5-HT modulation of I_h_ currents	5-HT suppresses I_h_ currents, reducing low-frequency resonance	Whole-cell patch clamp with 5-HT application	Development-dependent serotonergic modulation of frequency tuning	Tanaka et al. (2019)
Orexin A effect on I_NaP_	Enhances I_NaP_ density, shifts activation curve negatively	Whole-cell patch clamp with orexin A bath application	Increases firing frequency and resonance; links feeding/metabolism to oral motor control	Tanaka et al. (2021)
NPY effect	Depolarization, inward current induction, shortened AHP, increased spike and burst frequency	Whole-cell patch clamp with NPY bath application	Enhances excitability via Y1/Y5 receptors; links appetite signals to oral motor reflexes	Seki et al. (2020)

The table details their electrophysiological features, experimental approaches used to study them, functional roles in excitability and sensory processing, and the key references that provided these findings.

## 4 Functional roles of MTNs in mastication and sensorimotor integration

### 4.1 Encoding of proprioceptive signals from jaw muscles

As the only primary afferent fibers with cell bodies located within the CNS, MTNs encode tension changes from masseter spindles into spike frequency, thereby forming the sensory basis for jaw motor control (Kato et al., 1999). Clinically, resection of the trigeminal sensory root or Gasserian ganglion in patients with idiopathic trigeminal neuralgia abolishes the jaw-jerk reflex without altering masseter EMG activity, indicating that the afferent pathway of the jaw reflex projects via the sensory root to the Vmes rather than the motor root (Ongerboer de Visser, 1982). In the rat Vmes, paired whole-cell recordings have demonstrated that electrically coupled neuronal clusters respond with high sensitivity to simultaneous depolarization, functioning as coincidence detectors that selectively extract temporal correlations from inputs. This sensitivity is determined by both intercellular coupling strength and intrinsic excitability, particularly I_h_ currents, and it is dramatically enhanced by cGMP-dependent augmentation of I_h_ (Khakh and Henderson, 1998; Davoine and Curti, 2019). More recent studies have revealed that I_h_ currents dynamically modulate coupling coefficients in the hyperpolarized range, shortening the coincidence detection window and suggesting that electrical synchrony and sensory processing in Vmes circuits are dynamically regulated (Trigo et al., 2025). These findings indicate that MTNs encode jaw muscle proprioception with high temporal precision in real time, contributing to fine-tuning of masticatory rhythm and occlusal force through the interaction of intrinsic excitability and electrical coupling.

### 4.2 Crucial role in masticatory rhythmogenesis

The Vmes is a central component of the sensorimotor loops involved in mastication and occlusal control, with its neurons playing a pivotal role in rhythm generation by central pattern generators. MTNs display sustained rhythmic firing from developmental stages, with the stability of their bursts increasing with maturation (Wu et al., 2001). According to Pedroarena et al. (1999), rat MTNs exhibit rhythmic burst firing in response to depolarizing stimulation, contributing to the periodicity of jaw movements. This electrical activity suggests an intrinsic firing capability essential for generating repetitive patterns in mastication. Moreover, M-V neurons project their central axons to the Vmo, thereby forming a sensory–motor loop that synchronizes sensory input with motor output in real time (Luo and Dessem, 1996). This circuitry contributes to synchronous contraction of jaw muscles and ensures fine control of masticatory rhythm. Additionally, MTN cell bodies receive synaptic inputs from multiple brainstem regions and express a variety of neurotransmitter receptors (Copray et al., 1990), providing a structural substrate for integrating peripheral sensory inputs with central modulation. These findings highlight the functional significance of MTNs in the rhythmogenesis of mastication.

### 4.3 Reflex control: jaw-jerk reflex and its modulation

The jaw-jerk reflex, the fastest orofacial motor reflex, is triggered by MTNs and mediated monosynaptically via the Vmo to induce contraction of the masseter muscle. In both rats and cats, stimulation of MTN cell bodies elicits masseter EMG responses with conduction delays of only 2–3 ms, reflecting a high-speed loop in which sensory input and motor output are virtually inseparable (Ongerboer de Visser, 1982; Lund et al., 1984). This reflex is subject to dynamic modulation by neurotransmitters. Whole-cell recordings in brainstem slices have demonstrated that histamine depolarizes both MTNs and masseter motoneurons but suppresses MTNs via H1 receptors, thereby reducing synaptic efficiency of the jaw-closure reflex (Gemba et al., 2015). Conversely, behavioral studies in cats have revealed that environmental stimuli increasing arousal, such as loud white noise or predator exposure, enhance masseter reflexes via elevated monoaminergic activity, particularly that mediated by NE. This facilitation was abolished by the α1-blocker prazosin, confirming NE dependence (Stafford and Jacobs, 1990). Furthermore, click stimuli induce reflex suppression at approximately 20 ms and enhancement at 100–150 ms post-stimulus, indicating that central synaptic plasticity dynamically modulates reflex amplitude within specific time windows. Collectively, these findings demonstrate that the Vmes–Vmo loop undergoes antagonistic modulation: histaminergic suppression and NE-mediated facilitation finely tune jaw-jerk reflex gain according to behavioral state and arousal levels (Stafford and Jacobs, 1990).

### 4.4 Sensorimotor integration for coordinated jaw movements

MTNs receive spindle-derived inputs from jaw muscles and contribute to coordinated movements through multiple connections with the Vmo, cerebellum, and vestibular system. Recent studies have found that jaw muscle spindle afferents, via “common premotor neurons,” exert parallel influence on multiple orofacial motor nuclei, revealing a premotor divergence mechanism that facilitates spatiotemporal coordination of mastication, tongue, and facial muscles beyond simple reflex responses (Zhang et al., 2012). Immunohistochemical studies have further demonstrated that many trigeminal premotor neurons are glutamatergic, supporting the predominance of excitatory drive in premotor layers receiving Vmes inputs (Turman and Chandler, 1994). This circuit organization provides the anatomical substrate for rapid force distribution and synchronization during food manipulation and speech. Notably, anatomical pathways linking the cerebellum and oculomotor control nuclei enable integration of mastication with the tongue, swallowing, and fine orofacial movements required for articulation. Chen et al. (2022) demonstrated that projections from the Vmes to oculomotor and interstitial nuclei mediate masticatory–ocular coordination in response to head angle changes under gravity. This indicates that sensorimotor loops both regulate muscle contraction and enhance motor adaptability. Furthermore, it has been proposed that real-time modulation of sensory input via the cortex–Vmes–Vmo loop contributes to precision in timing and force control during food comminution (Masuda et al., 1997).

### 4.5 Electrophysiological similarities and differences between MTNs and trigeminal motor neurons (TMNs)

Mesencephalic trigeminal neurons process proprioceptive inputs from the jaw-closing muscles and periodontal ligaments, whereas TMNs directly generate motor output to the mandibular muscles. Although both neuron types participate in the formation of masticatory rhythms and jaw movement control, they display distinct yet complementary electrophysiological profiles. A major similarity is their intrinsic excitability and plastic firing patterns, which are dynamically regulated by neuromodulators. MTNs are characterized by high input resistance and low firing threshold, and their rhythmic firing depends on Nav1.6-mediated I_NaP_ and I_NaR_ currents that confer subthreshold resonance and burst generation (Enomoto et al., 2007). Similarly, TMNs exhibit repetitive firing based on Na^+^ and Ca^2+^ currents, producing rhythmic motor discharges that drive jaw-closing movements during mastication (Nishimura et al., 2018). Both neuronal populations are also subject to modulation by 5-HT, orexin, and NE, which alter membrane excitability by adjusting threshold potentials and after-depolarization amplitudes according to the behavioral state (Tanaka et al., 2020). These intrinsic conductance and neuromodulatory mechanisms allow close sensory–motor coupling within the masticatory central pattern generator.

IConversely, clear differences exist between MTNs and TMNs. As primary sensory neurons, MTNs possess Nav1.6-dependent Na^+^ currents in combination with D-type K^+^ currents and connexin-36–mediated gap junctions (Curti et al., 2012; Dapino et al., 2023). These features enable strong electrical coupling among MTNs, synchronizing their activity to enhance temporal precision of proprioceptive signaling. Such synchronous firing stabilizes sensory feedback during rhythmic jaw movements and contributes to fine adjustment of bite force (Curti et al., 2012; Kolta et al., 2007; Davoine and Curti, 2019). By contrast, TMNs exhibit specialization for motor output. *α*-Type TMNs generate fast, powerful contractions of jaw-closing muscles, whereas *γ*-type TMNs regulate muscle spindle sensitivity. These subtypes differ in firing threshold and after-depolarization properties (Nishimura et al., 2018). Thus, TMNs are optimized for stable and sustained motor discharge required for precise and forceful orofacial movements.

In summary, MTNs provide synchronized and high-precision sensory coding, whereas TMNs ensure continuous and controlled motor output, forming a complementary sensorimotor partnership. Comparative electrophysiological analysis of these neurons is essential for elucidating the neural mechanisms underlying masticatory rhythmogenesis and sensorimotor coordination, and it might also yield valuable insights into early pathophysiological changes in disorders such as amyotrophic lateral sclerosis and masticatory dysfunction.

## 5 Electrophysiology in disarray: implications for dysfunction and disease

### 5.1 Relationship between clenching and MTNs

Electrophysiological hyperactivity of primary afferent neurons has been proposed as a key mechanism underlying masseter hypertonicity in bruxism and temporomandibular disorders (TMDs). In a chronic jaw muscle myalgia model induced by acidic saline injection, MTNs, which contain the cell bodies of muscle spindle afferents, exhibited an enhancement of I_NaP_ currents and generated ectopic firing mediated by subthreshold membrane oscillations (Sas et al., 2024). This hyperexcitability was further amplified by astrocyte-derived S100β, which reduced extracellular Ca^2+^ levels, suggesting that neuron–glia interactions play a crucial role in muscle spindle afferent overactivity (Gaudel et al., 2025).

Beyond astrocytes, accumulating evidence suggests that other glial cell types may also modulate sensory neuron excitability under pathological conditions. Microglia can influence neuronal firing through activity-dependent release of pro-inflammatory mediators and neuromodulatory factors, including cytokines and neurotrophic signals that regulate synaptic strength and network excitability (Klapal et al., 2016). Oligodendrocytes and oligodendrocyte precursor cells (OPCs), in addition to supporting axonal conduction via myelination, are now recognized to impact neuronal circuit function by regulating action potential propagation and engaging in bidirectional interactions with neurons that can affect circuit excitability and timing (Yamazaki, 2019). Although the precise roles of these glial populations in MTN dysfunction remain to be elucidated, such multicellular interactions are likely to shape the excitability landscape of muscle spindle afferents and contribute to the persistence of masticatory muscle hyperactivity.

At the central level, chronic restraint stress has been demonstrated to increase MTN excitability, accompanied by enhanced VGLUT1-positive excitatory inputs to the Vmo. This leads to persistent elevation of masseter electromyographic activity. shRNA-mediated silencing of VGLUT1 expression in the Vmes abolished this overactivity, indicating that stress-induced MTN hyperexcitability constitutes a central mechanism in TMD pathogenesis (Zhao et al., 2022). Thus, hyperactivity at both peripheral (muscle spindle afferents) and Vmes levels appears to form a core neurophysiological substrate underlying abnormal masseter contraction and pain hypersensitivity observed in bruxism and TMDs.

Clenching, encompassing tooth grinding and sustained jaw contraction, is not a simple peripheral phenomenon arising from occlusal abnormalities; instead, it is a complex motor behavior resulting from a breakdown of coordination within neural circuits integrating masticatory muscle activity, proprioceptive input, and central modulation (Uchima Koecklin et al., 2023). MTNs, which occupy the central position in this system, receive proprioceptive input from jaw-closing muscle spindles and periodontal ligaments and project centrally via their axons to motor nuclei such as the Vmo. As intracerebral primary afferent neurons, MTNs respond sensitively to tooth contact and tension changes, dynamically regulating masticatory muscle activity and reflex gain (Giovanni and Giorgia, 2021).

Neurophysiologically, a positive feedback loop comprising tension loading of the masticatory muscles and periodontal ligaments, excitation of MTNs, facilitation of Vmo output, and sustained masseter hyperactivity has been proposed. In chronically stressed mice, both MTN excitability and VGLUT1-positive projections are enhanced, whereas VGLUT1 suppression reduces masseter overactivity (Zhao et al., 2022). Furthermore, MTNs are interconnected with arousal and emotional circuits. Through periodontal ligament input, MTNs can activate the ascending reticular activating system and the hypothalamic orexin network (Pavlou et al., 2024). In sleep bruxism, persistent activation of the MTN–ascending reticular activating system pathway is believed to contribute to sustained muscle tone and increased reflex gain (Giovanni and Giorgia, 2021). In addition, studies using occlusal disharmony models have revealed that activation of the lateral habenula–MTN pathway links anxiety to masseter overactivity, indicating that emotional stress can enhance masticatory muscle tension through MTN excitation (Liu et al., 2019; Zhao et al., 2022). From a reflex control perspective, MTNs play an essential role as the afferent limb of the jaw-stretch and masseter-stretch reflexes. In patients with bruxism, an enhancement of masseter inhibitory reflex suppression has been observed (Luco, 2017), suggesting that altered MTN function forms the neural basis for persistent clenching.

Clinically, elucidation of the MTN–Vmo circuit and its interconnections with the arousal, emotional, and stress–muscle tension systems provide a new conceptual framework for understanding and treating bruxism and TMDs (Uchima Koecklin et al., 2023). Therapeutic strategies that suppress MTN excitability, such as neuromodulation, modulation of muscle spindle responsiveness, stress management, and reduction of tooth contact frequency through occlusal splints, represent rational, multidimensional interventions. Future studies should aim to visualize MTN activity in humans and evaluate whether targeted neuromodulatory approaches toward MTN function can ameliorate the pathophysiology of clenching and related orofacial disorders.

### 5.2 Channelopathies and synaptopathies: molecular roots of Vmes dysfunction

MTNs sustain rhythmic bursting through Nav1.6-dependent I_NaP_, I_NaP_, and I_h_ currents. In mice lacking Nav1.6 channels, I_NaP_ currents are reduced by 39%, and I_NaR_ currents are suppressed by 76%, leading to the loss of subthreshold oscillations, impedance resonance, and both spontaneous and evoked rhythmic firing (Enomoto et al., 2006, 2007). Similarly, in the SOD1G93A amyotrophic lateral sclerosis (ALS) model, MTNs display early downregulation of Nav1.6 expression, resulting in irregular and arrested firing patterns. Computational modeling suggests that such sensory disruption can shift trigeminal motoneurons into an autonomous firing mode, producing fasciculations and abnormal masticatory rhythms. These findings identify Nav1.6 channelopathy as an early, selective defect within the Vmes circuit, potentially triggering bruxism, TMDs, and ALS-associated motor disturbances (Seki et al., 2019).

In addition, glutamatergic synaptic dysfunction (synaptopathy) within the Vmes leads to profound network failure. In sudden infant death syndrome models, immature glutamatergic synapses between the Vmes and reticular circuits delay arousal reflexes, thereby increasing the risk of fatal apnea (Andrisani and Andrisani, 2015). Given that long-term potentiation is essential for brainstem motor circuit maturation, Vmes synaptopathies might extend beyond mastication to affect vital life-sustaining reflexes.

Together, Nav1.6 channelopathies and glutamatergic synaptopathies represent dual molecular disruptions that destabilize Vmes circuitry, precipitating a spectrum of orofacial motor abnormalities and autonomic dysfunction. Supporting this, Rett syndrome models (Mecp2^R168X/Y^, Mecp2^Bird/Y^) exhibit intrinsic membrane abnormalities in MTNs in addition to locus coeruleus dysfunction, with lowered thresholds driving enhanced firing. Such hyperexcitability can disturb brainstem excitatory–inhibitory balance, contributing to respiratory and motor dysfunction (Johnson et al., 2016; Zhong et al., 2017).

Of further relevance, the locus coeruleus, an early degenerative target in Alzheimer’s disease, is anatomically and functionally interconnected with the Vmes. Peripheral afferent signals relayed via MTNs, such as those induced by periodontal disease or bacterial infection, can accelerate locus coeruleus neurodegeneration, implicating MTNs as conduits linking oral pathology to Alzheimer’s disease-related neurodegeneration (Pisani et al., 2023).

### 5.3 Electrophysiological comparison of primary afferent neurons: MTNs, TG neurons, and DRG neurons

MTNs, TG neurons, and DRG neurons are primary sensory neurons that transmit afferent information to the CNS. Although these neurons share a common role as first-order afferents, their electrophysiological properties differ according to their functional specialization. A fundamental similarity among these three neuron types is that voltage-gated sodium channels form the basis of action potential generation and repetitive firing (Liu et al., 2019). The combination of tetrodotoxin-sensitive and tetrodotoxin-resistant Na^+^ channels determines threshold levels, firing frequency, and the ability to sustain repetitive discharges (Liu et al., 2022; Vasylyev et al., 2023; Huff et al., 2025). In addition, K^+^ channels, particularly the delayed rectifier K^+^ channel family responsible for repolarization and afterhyperpolarization, together with inwardly rectifying K^+^ currents and I_h_ currents, regulate membrane excitability, firing adaptation, and rebound activity. These conductances constitute the shared core mechanisms of excitability control in primary sensory neurons (Liu et al., 2017, 2022; Vasylyev et al., 2023; Huff et al., 2025).

In contrast, clear differences have been observed among MTNs, TG neurons, and DRG neurons. TG and DRG neurons comprise heterogeneous subtypes that process nociceptive, thermal, and mechanical sensory inputs from peripheral tissues, including small-diameter C-fiber, medium Aδ-fiber, and large Aβ-fiber neurons. TG neurons display high expression of Nav1.7 channels (tetrodotoxin-sensitive), which lower the activation threshold and facilitate high-frequency firing. In experimental pain models, overexpression of Nav1.7 channels leads to pathological hyperexcitability. In DRG neurons, I_h_ currents are prominently observed. Recent studies have suggested that I_h_ currents can exert inhibitory effects by increasing the rheobase (minimum current required for firing), thereby reducing firing probability in small-diameter DRG neurons (Liu et al., 2022; Vasylyev et al., 2023; Huff et al., 2025). Moreover, DRG neurons display large variability in channel expression, including Nav1.8 and Nav1.9 (tetrodotoxin-resistant), contributing to pronounced plasticity in firing patterns and voltage responses under chronic pain and neuropathic conditions.

Both TG and DRG neurons exhibit peripheral neuronal specialization characterized by subtype diversity and adaptive firing plasticity. TG neurons are specialized for sensory input from the craniofacial region, whereas DRG neurons process sensory information from the trunk and limbs. Their molecular expression profiles reflect this regional distinction, displaying “craniofacial-specific” versus “somatic–peripheral-specific” gene patterns. Contrarily, MTNs function as part of a centrally located proprioceptive system that governs rhythmic and synchronous reflex control. Electrophysiologically, MTNs are distinguished by I_NaP_ currents, Ires, gap-junction coupling, and resonant membrane properties, features that support rhythmic and synchronized activity. In comparison, TG and DRG neurons rely on the Nav1.7/Nav1.8/HCN channel families, exhibit adaptive firing patterns, and display marked excitability plasticity (Lopes et al., 2017).

Thus, although MTNs, TG neurons, and DRG neurons share a common framework as primary afferents, their electrophysiological mechanisms of excitability control differ substantially because of variations in role, circuit organization, and ion channel expression. Understanding these distinctions provides valuable insight into the pathophysiological basis of masticatory reflexes, facial pain, and peripheral neuropathic pain, and may aid in identifying novel therapeutic targets for sensory and orofacial disorders.

### 5.4 Age-related alterations in electrophysiological properties

Age-related changes in MTN function exert profound effects on the regulation of mastication and sensory stability. In aged mice (25–31 months), MTN numbers are significantly reduced, with marked nuclear enlargement and lipofuscin accumulation, whereas adjacent masseter motoneurons display loss of Nissl bodies (Sturrock, 1987). Beyond natural senescence, tooth loss represents an additional driver of MTN degeneration. In C57BL/6 J mice, molar extraction led to a significant reduction in Piezo2 expression within MTNs, accompanied by ATF3- and caspase-3–positive cells indicative of neuronal death (Dhar et al., 2021).

Simultaneously, trigeminal motoneurons exhibited TDP-43–positive neuronal cytoplasmic inclusions, suggesting that Vmes degeneration can propagate into lower motoneuron systems (Dhar et al., 2021). Moreover, in 3 × Tg-AD Alzheimer’s disease model mice, tooth extraction induced the accumulation of cytotoxic Aβ42 in MTNs and subsequent neuronal death, which extended to degeneration of the locus coeruleus, thereby exacerbating hippocampal neuronal loss and accelerating spatial memory decline (Goto et al., 2020).

Together, these findings suggest that aging and tooth loss initiate a Vmes-centered cascade of neurodegeneration, with consequences extending beyond impaired mastication to cognitive decline (Table 3).

**Table 3:** Pathophysiological implications of MTN dysfunction across various conditions and diseases, including bruxism, TMDs, ALS, and aging-related changes.

**Condition/disease**	**Reported MTN dysfunction**	**Consequences**	**Supporting evidence**	**Potential therapeutic target**	**Key references**
Bruxism	Hyperexcitability, irregular firing	Involuntary jaw clenching	Clinical EEG + animal studies	Ion channel modulators	Sas et al. (2024) and Gaudel et al. (2025)
TMD	Abnormal proprioceptive feedback	Impaired jaw reflex; pain	Human studies	Sensory–motor rehabilitation	Zhao et al. (2022)
ALS	Nav1.6 downregulation; irregular firing	Masticatory rhythm disruption	SOD1 model electrophysiology	Neuroprotective strategies	Seki et al. (2019) and Kitaoka et al. (2023)
Aging/tooth loss	Reduced Piezo2 expression; neuronal loss	Decline in mastication; cognitive impairment	Rodent models	Proprioceptive restoration; implant therapy	Sturrock (1987), Florez-Paz et al. (2016), Dhar et al. (2021), and Goto et al. (2020)
Rett syndrome	Intrinsic membrane hyperexcitability of MTN; decreased spike threshold	Disrupted excitatory/inhibitory balance in brainstem circuits; respiratory and motor dysfunction	Mecp2 mutant mice (Mecp2R168X/Y, Mecp2Bird/Y) electrophysiology	Modulation of excitability; targeting monoaminergic balance	Johnson et al. (2016) and Zhong et al. (2017)
Alzheimer’s disease (AD)	MTN degeneration and accumulation of Aβ42 after tooth loss; trans-synaptic spread to locus coeruleus	Hippocampal neuron loss; spatial memory decline; impaired mastication	3 × Tg-AD mouse model; tooth extraction experiments	Oral health intervention; prevention of peripheral inflammation	Goto et al. (2020) and Pisani et al. (2023)
Sudden infant death syndrome (SIDS)	Immature glutamatergic synapses between Vmes and reticular formation; impaired arousal reflex	Delayed or absent arousal from hypoxia; increased risk of lethal apnea	SIDS-like neonatal rodent models; glutamatergic synaptogenesis studies	Enhancement of synaptic maturation; brainstem excitability support	Andrisani and Andrisani (2015)

The table summarizes the reported abnormalities in MTN activity, their physiological and behavioral consequences, supporting clinical or experimental evidence, potential therapeutic targets, and representative references.

## 6 Vmes neurons as therapeutic targets

The Vmes consists of primary afferent neurons that transmit proprioceptive information from the masticatory muscles to the brainstem, playing a crucial role in the control of mastication. In animal models, psychological stress has been demonstrated to increase the excitability of MTNs, resulting in masseter muscle hyperactivity (Zhao et al., 2022). In this study, mice exposed to restrain stress for 14 days exhibited both anxiety-like behaviors and hyperactivity of the masseter muscle, accompanied by heightened electrophysiological excitability of MTNs. Moreover, VGLUT1-positive glutamatergic projections from the Vmes to the Vmo were enhanced, and shRNA-mediated suppression of VGLUT1 by attenuated these pathological changes, suggesting that MTNs are deeply involved in the central mechanisms of TMDs.

In addition, transcranial direct current stimulation has been reported to indirectly modulate the Vmes and sensory nuclei via trigeminal branches (Majdi et al., 2024). In this rat experiment, direct current stimulation of the trigeminal nerve transiently increased MTN spiking activity, which was abolished by anesthetic blockade, confirming that Vmes modulation can be achieved through peripheral nerve pathways. Furthermore, in ALS model mice, disrupted masticatory rhythm and reduced MTN firing were also observed (Seki et al., 2019; Kitaoka et al., 2023; Seki et al., 2023), suggesting that Vmes dysfunction contributes to feeding disturbances in neurodegenerative diseases. Collectively, these findings indicate that the Vmes is a central mechanism involved in stress, neurodegeneration, and neuromodulation, thereby providing insight into the pathophysiology and potential therapeutic approaches for human TMDs and ALS.

Recently, the plasticity of MTN activity and its functional interactions with multiple systems have drawn attention to the potential of these neurons as therapeutic targets. For example, Zhao et al. (2022) demonstrated that chronic restraint stress enhanced MTN excitability, excessively activating the Vmo through VGLUT1-positive glutamatergic projections, thereby inducing masseter hyperactivity. Moreover, shRNA interference targeting VGLUT1 in the Vmes suppressed neuronal hyperexcitability and abnormal muscle activity, suggesting that gene interference therapy targeting the Vmes could ameliorate TMD pathology (Zhao et al., 2022).

Fortin et al. (2021) reported that the Vmes expresses melanocortin 4 receptor, and that local administration of the melanocortin 4 receptor agonist melanotan II significantly reduced food intake and body weight. Furthermore, chemogenetic activation of proopiomelanocortin neuronal projections from the nucleus tractus solitarius to the Vmes also elicited feeding suppression, suggesting that the Vmes is part of a central circuit regulating metabolism. In addition, neuropeptides such as NPY, orexin, and 5-HT modulate MTN membrane potential, firing frequency, and I_h_ and Na^+^ currents, thereby playing important roles in rhythmic mastication and oromotor control (Tanaka et al., 2019; Seki et al., 2020; Tanaka et al., 2021; Yamada et al., 2021).

Importantly, MTNs also receive innervation from nitric oxide synthase-containing fibers and respond electrophysiologically to nitric oxide, suggesting modulation of excitability and synaptic transmission via the nitric oxide–cGMP pathway (Pose et al., 2003). This gaseous messenger-based plasticity offers multiple intervention points, combining pharmacological approaches (nitric oxide synthase inhibitors, nitric oxide donors, soluble guanylyl cyclase modulators) with neuromodulatory devices. Taken together, MTNs hold significant potential as novel therapeutic targets for TMDs, feeding disorders, and neurodegenerative diseases such as ALS. Future studies are expected to test integrated strategies that simultaneously target multiple modules, including VGLUT1-mediated projections, peptide modulation, and nitric oxide signaling.

## 7 Conclusion

As revealed in this review, mesencephalic trigeminal neurons (MTNs) are being redefined as electrophysiologically dynamic and functionally diverse central nodes that play a pivotal role in the timing and force control of mastication. Through advanced firing patterns, including tonic activity, bursting, and membrane resonance, MTNs precisely regulate masticatory rhythms and reflex movements, integrating intrinsic membrane properties with neuromodulatory influences.

Chronic stress has been shown to induce MTN hyperexcitability, leading to abnormal activation of masseter motoneurons via VGLUT1-positive glutamatergic projections and resulting in masseter hyperactivity and temporomandibular disorders (TMDs) in animal models (Zhao et al., 2022).

Importantly, this pathological state can be ameliorated by gene interference targeting the Vmes, highlighting the potential of central intervention-based therapeutic strategies for functional masticatory disorders.

Beyond mastication, the Vmes has emerged as a multifunctional regulatory hub involved in feeding behavior and metabolic control. It functions as part of novel central networks mediated by melanocortin 4 receptor signaling and the nucleus tractus solitarius–proopiomelanocortin pathway, positioning the Vmes as a neuromodulatory target for obesity and feeding disorders (Fortin et al., 2021). Furthermore, reduced MTN firing and disrupted masticatory rhythms observed in ALS model mice suggest that Vmes dysfunction may contribute to feeding impairments in motor neuron diseases (Kitaoka et al., 2023).

Despite these advances, important challenges remain in fully defining the molecular substrates underlying MTN excitability. While a broad range of ionic conductances has been functionally characterized, the precise molecular identities of several channels remain unresolved, reflecting historical and technical constraints. Where convergent electrophysiological, genetic, and molecular evidence is available, specific channel subtypes have been identified; however, other conductances, such as ATP-evoked P2X receptor-mediated currents, exhibit atypical properties that suggest the presence of MTN-specific channel assemblies or regulatory mechanisms.

Accordingly, this review has emphasized functionally robust ionic mechanisms while assigning molecular identities only when supported by direct experimental evidence. Future advances in single-cell transcriptomics, spatial molecular profiling, and cell-type-specific functional analyses will be essential for refining the molecular taxonomy of MTN ion channels and for translating mechanistic insights into targeted neuromodulatory and molecular therapeutic strategies for orofacial and neurodegenerative disorders.
